# Lumacaftor/ivacaftor in cystic fibrosis: effects on glucose metabolism and insulin secretion

**DOI:** 10.1007/s40618-021-01525-4

**Published:** 2021-02-13

**Authors:** C. Colombo, A. Foppiani, A. Bisogno, S. Gambazza, V. Daccò, E. Nazzari, A. Leone, A. Giana, A. Mari, A. Battezzati

**Affiliations:** 1 Cystic Fibrosis Center, Fondazione IRCCS Cà Granda Ospedale Maggiore Policlinico, Via Commenda 9, 20122 Milan, Italy; 2 Department of Pathophysiology and Transplantation, University of Milan, Milan, Italy; 3 International Center for the Assessment of Nutritional Status (ICANS), Department of Food Environmental and Nutritional Sciences (DeFENS), University of Milan, Milan, Italy; 4 Direzione delle Professioni Sanitarie, Fondazione IRCCS Ca’ Granda Ospedale Maggiore Policlinico, Milan, Italy; 5 National Research Council (CNR), Institute of Neuroscience, Padua, Italy

**Keywords:** Phe508del, Lumacaftor/ivacaftor, Glucose tolerance, OGTT

## Abstract

**Purpose:**

The question whether the new cystic fibrosis transmembrane conductance regulator (CFTR) modulator drugs aimed at restoring CFTR protein function might improve glucose metabolism is gaining attention, but data on the effect of lumacaftor/ivacaftor treatment (LUMA/IVA) on glucose tolerance are limited. We evaluated the variation in glucose metabolism and insulin secretion in CF patients homozygous for Phe508del CFTR mutation after one-year treatment with LUMA/IVA in comparison to patients with the same genotype who did not receive such treatment.

**Methods:**

We performed a retrospective case–control study on 13 patients with a confirmed diagnosis of CF, homozygous for the Phe508del CFTR mutation, who received LUMA/IVA for one year (cases) and 13 patients with identical genotype who did not receive this treatment (controls). At the beginning and conclusion of the follow-up, all subjects received a modified 3 h OGTT, sampling at baseline, and at 30 min intervals for plasma glucose, serum insulin, and c-peptide concentrations to evaluate glucose tolerance, and quantify by modeling beta-cell insulin secretion responsiveness to glucose, insulin clearance and insulin sensitivity.

**Results:**

LUMA/IVA did not produce differences in glucose tolerance, insulin secretory parameters, clearance and sensitivity with respect to matched controls over one-year follow-up.

**Conclusion:**

We found no evidence of improvements in glucose tolerance mechanisms in patients with CF after one-year treatment with LUMA/IVA.

## Introduction

Cystic fibrosis-related diabetes (CFRD) is probably the most clinically relevant extra-pulmonary complication of cystic fibrosis (CF) and is associated with increased morbidity and mortality in affected individuals.

The pathophysiology of CFRD is complex and results from insulin and glucagon deficiency (due to decreased islet cell mass and β-cell dysfunction), intermittently exacerbated by insulin resistance attributable to the chronic inflammatory pulmonary state that characterizes these patients. A possible role of the expression of the cystic fibrosis transmembrane conductance regulatory (CFTR) protein in pancreatic islet beta cells and CFTR defects in contributing to abnormalities in insulin secretion has also recently emerged [1].

At present, the gold standard for CFRD screening and diagnosis is the oral glucose tolerance test (OGTT), that is recommended annually by the Cystic Fibrosis Foundation and the American Diabetes Association’s guidelines in all patients with CF older than 10 years of age [2].

The incidence of CFRD increases with age. While CFRD is rarely present in children (less than 2%) [3], insulin secretory defects in CF were found to be extremely frequent, also in normotolerant CF patients who compensate with an increased insulin sensitivity [4]. Such defects worsen with advancing age, and CFRD prevalence progressively increases up to 50% of individuals 30 years of age and older [3]. The cause of the insulin secretory defects could be either related to progressive fibrosis of the exocrine pancreas, local inflammation or to an intrinsic islet dysregulation caused by the basic defect. In addition, glucose tolerance is highly variable in CF patients as a result of fluctuating levels of insulin resistance due to acute and chronic infections since the first years of life [5].

The question whether new CFTR modulator drugs aimed at restoring CFTR channel function might improve glucose metabolism is gaining attention. Early observations referred to Ivacaftor treatment in limited numbers of individuals with the G551D mutation indicated beneficial effects spanning from resolution of CFRD after 13 months of treatment in a single 25-year-old patient, to improvement in glucose tolerance category in 4 out of 5 examined patients [6, 7]. More recent analyses of two independent CF registries (the US and UK cystic fibrosis registries) showed favorable trends in CFRD with Ivacaftor treatment vs comparators, with lower prevalence of CFRD with time, suggesting that this treatment may improve glycemic control [8, 9]. However, the G551D mutation only accounts for 4–5% of CF cases worldwide.

Lumacaftor/ivacaftor (LUMA/IVA, Orkambi^®^), approved for CF patients homozygous for the Phe508del CFTR mutation, includes a medication correcting intracellular processing of CFTR (the corrector lumacaftor) with one that increases the open probability of the abnormal CFTR channel (the potentiator ivacaftor). While this CFTR modulator combination targets the basic defect in CF, it only mildly improves CFTR channel activity [3]. In in vivo, this combination has led to a slight increase in forced expiratory volume in 1 s (FEV_1_) percent predicted, reduction in pulmonary exacerbation risk, improvement in weight and pulmonary symptoms compared to placebo [10], whereas the effects on sweat chloride were small [11].

There are limited data on the effect of LUMA/IVA treatment on glucose tolerance. A small study showed that in five CF patients homozygous for the Phe508del CFTR mutation, treatment for 6–8 weeks had no consistent impact on glucose tolerance and insulin secretion evaluated by means of OGTT and intravenous glucose tolerance tests [12]. Similarly, no changes in glucose concentrations 1 and 2 h after OGTT or during continuous glucose monitoring were observed in 9 young CF patients [13]. On the other hand, one recent study on 40 patients with CF did show a better glucose tolerance, as assessed by a reduction in 1 h and 2 h OGTT glycemia, after one-year treatment with LUMA/IVA [14]. However, glucose tolerance and insulin secretion were not fully characterized in those studies, which in addition did not account for the natural fluctuations of OGTT-derived parameters in CF [15].

Our aim was therefore to evaluate the variation in glucose metabolism and insulin secretion in CF patients homozygous for Phe508del CFTR mutation after one-year treatment with LUMA/IVA in comparison to patients with the same genotype who did not receive such treatment.

## Materials and methods

### Study design

This was a retrospective case–control study on patients with a confirmed diagnosis of CF, homozygous for the Phe508del CFTR mutation, regularly followed up at the Cystic Fibrosis Centre of Fondazione IRCCS Ca’ Granda, Ospedale Maggiore Policlinico. Data were collected from hospital records of all patients with CF who had been treated over a 17-year-period (2003–2020). The study was approved by the Milano Area 2 ethical committee (*n*. 452, 556 and 777) and written consent was obtained from each patient or subject after full explanation of the purpose and nature of all procedures used.

### Subject selection

Since 2003, all patients with CF older than 10 years have undergone clinical and laboratory assessment, including an annual OGTT, and were offered to participate in a study of the natural history of glucose tolerance defects in CF. During follow-up visits as outpatients, those who consented underwent a modified OGTT as described below if they had been clinically stable in the previous 3 weeks (absence of major clinical events including pulmonary exacerbations, no change in their habitual treatment regimen including introduction of antibiotics or steroids) and if they had not received a CFRD diagnosis or treatment with insulin or oral hypoglycemic agents in the previous 6 months.

For the purpose of this study, we enrolled CF patients homozygous for Phe508del mutation who agreed to participate in the study of glucose tolerance defects in CF, had been screened for LUMA/IVA but had not yet started the treatment. Prior to and after 1 year of treatment, patients—referred from here on as cases—performed a basic anthropometric assessment, OGTT, and spirometry according to the follow-up protocol described above. In cases, LUMA/IVA was administered at the dose of 800 mg lumacaftor and 500 mg ivacaftor daily in two divided doses.

Controls were selected from an available group of 98 CF patients homozygous for the Phe508del CFTR mutation and 377 observations. These represented the historical naive controls who had received at least two modified OGTTs spaced one year apart, participating in the study of glucose tolerance defects before the advent of LUMA/IVA. Cases and controls were required to have the same genotype and follow-up time (distance between first and second examination).

### Anthropometric assessment

Weight and height were collected at each visit, body mass index (BMI) was calculated as weight in kilograms divided by the square of the height in meters.

### Oral glucose tolerance test

OGTT was performed with a modified protocol already used in CF [16]. OGTTs were performed sampling at baseline and at 30 min intervals for 3 h for plasma glucose, serum insulin, and C-peptide concentrations. In controls, OGTT was repeated as part of their standard of care monitoring. All the OGTTs (1.75 g/kg, maximum 75 g) were performed in fasting condition since at least 8 h. Plasma glucose was measured on fluoride plasma samples (Glucoquant; Roche/Hitachi analyzer; Roche Diagnostics), and the other analytes were measured by commercial assays (ECLIA-Cobas C6000; Roche Diagnostics).

### Pulmonary function assessment

Spirometry was performed according to the American Thoracic Society (ATS) and European Respiratory Society (ERS) guidelines [17]. FEV_1_ was expressed as percentage of the reference values [18].

### Analysis and modeling of oral glucose tolerance test

Glucose tolerance classification followed the 2018 International Society for Pediatric and Adolescent Diabetes (ISPAD) guidelines [2]. Patients were then clustered in 3 classes: normal glucose tolerance (NGT), impaired glucose tolerance (IGT, including impaired fasting, indeterminate and impaired glucose tolerance) and CFRD (including CFRD with and without fasting hyperglycemia). β-cell function was assessed from OGTT using a model that describes the relationship between insulin secretion and glucose concentration [19]. Insulin clearance was calculated during the OGTT as the ratio between the integral of insulin secretion and that of insulin concentration.

### Statistical analysis

Controls were obtained with a 1:1 optimal ratio matching based on the propensity score [20] estimated via the logistic regression that predicts the probability of being assigned to the treatment group conditional on baseline covariates [21]. The propensity score was based on age, sex, BMI, and FEV_1_. Balance between the two groups was evaluated with the standardized mean difference (SMD) of the matching variables.

A one-way analysis of covariance (ANCOVA) was performed on selected outcomes obtained from OGTT at one-year follow-up to evaluate the treatment effect whilst controlling for baseline values. Levene’s test and normality checks were carried out and the assumptions met.

Descriptive statistics are reported as mean (standard deviation) or count (fraction). Changes in baseline and follow-up glucose tolerance categories between cases and controls were evaluated by two-sample McNemar test. For all analyses, *P* values were two-sided and *P* < 0.05 was considered statistically significant. Analyses were performed using the open-source software R, version 4.0.2 [22], with package MatchIt added [23].

## Results

Thirteen cases and 13 matched controls were enrolled: the baseline characteristics of the two groups are reported in Table 1. Besides sex, which was perfectly matched, the other matching variables obtained an SMD between 0.1 and 0.2. The majority of patients were adults (10/13 cases and 11/13 controls). In each group, only 1 patient was underweight (adults with BMI < 18.5) and most patients (7/13 cases and 9/13 controls) had a normal glucose tolerance.

**Table 1:** Baseline characteristics of the two groups of patients with CF

	Cases	Controls	SMD
*N*	13	13	
Age (years)	21.03 (5.08)	21.71 (4.12)	0.148
Female sex	8 (61.5%)	8 (61.5%)	< 0.001
BMI (Kg/m^2^)	20.66 (2.94)	20.96 (1.79)	0.124
Pancreatic insufficiency	11 (100.0%)	12 (92.3%)	
FEV_1_ (% predicted)	81.89 (20.13)	85.45 (24.31)	0.160

Data are presented as mean (SD) or count (fraction).

*SMD* standardized mean difference

Table 2 presents the findings from ANCOVA. After adjustment by baseline level as covariate, only basal glucose after one year was significantly different between cases and controls, with *F* (1.23) = 5.086, *P* = 0.034, indicating significant treatment effect. Fasting glycemia resulted significantly higher by ~ 9 mg/dL in treated patients after 1 year of LUMA/IVA treatment, although still in the normal range. No evidence of statistically significant variation due to LUMA/IVA treatment was detected in glucose metabolism and insulin secretion variables. Table 3 also shows that distribution among the different glucose tolerance categories did not significantly change after 1 year of follow-up (*P* = 0.907). The 2 patients with CFRD treated with CFTR modulators persisted to be diabetic after 1 year of treatment, and one normotolerant patient became intolerant; among controls, one patient with reduced glucose tolerance at baseline developed CFRD. Only 1 of the naive patients with CFRD at the beginning of the study was diabetic at the end of the study, while the other 3 patients shifted between IGT and CFRD.

**Table 2:** Adjusted and Unadjusted Means of OGTT at one-year follow-up for cases and controls

	Group	Unadjusted Means		Adjusted mean	Adjusted mean difference with 95% CI
		Baseline	Follow-up		
BMI (Kg/m^2^)	Cases	21.0 (1.79)	21.6 (1.65)	21.5 (0.387)	0.5 [− 0.6–1.6]
	Controls	20.7 (2.94)	20.9 (2.64)	21 (0.387)	
FEV_1_ (% predicted)	Cases	85.5 (24.3)	85.2 (23.0)	85.6 (3.8)	4.5 [− 7.8–16.8]
	Controls	81.9 (20.1)	81.7 (26.9)	81.1 (4.5)	
Basal glucose (mg/dL)	Cases	87.2 (16.2)	89 (10.4)	86 (2.7)	8.7 [0.7–16.7]
	Controls	77.2 (12.8)	74.2 (14.6)	77.2 (2.7)	
120 min glucose (mg/dL)	Cases	123 (49)	131 (72)	136 (14)	− 6 [− 48–36]
	Controls	135 (59)	148 (68)	142 (14)	
Glucose AUC	Cases	7.54 (1.81)	7.72 (2.61)	7.79 (0.44)	− 0.03 [− 1.35–1.29]
	Controls	7.67 (1.83)	7.89 (2.05)	7.82 (0.46)	
β-cell glucose sensitivity (pmol × min^−1^ × m^−2^ × mM^−1^)	Cases	69.2 (51.3)	56.2 (33.0)	52.7 (6.1)	− 6.1 [− 24.7–12.4]
	Controls	55.6 (35.2)	55 (25.1)	58.9 (6.4)	
30 min C-peptide (ng/mL)	Cases	4.3 (2.2)	3.7 (1.4)	3.4 (0.3)	0.0 [− 0.9–0.9]
	Controls	3.2 (1.5)	3.1 (1.3)	3.4 (0.3)	
30 min insulin (µU/mL)	Cases	40 (30)	25 (16)	23 (4)	4 [− 8–16]
	Controls	20 (16)	17 (10)	19 (3)	
OGTT insulin clearance (L × min^−1^ × m^−2^)	Cases	1.27 (0.35)	1.75 (0.69)	2.13 (0.55)	− 0.14 [− 1.88–1.60]
	Controls	1.74 (0.54)	2.68 (2.73)	2.27 (0.57)	
2 h OGIS (ml × min^−1^ × m^−2^)	Cases	439 (56)	458 (49)	460 (37)	− 67 [− 170–36]
	Controls	527 (74)	542 (87)	527 (24)	

Standard deviations for unadjusted means and standard errors for adjusted mean

**Table 3:** Glucose tolerance categories for cases and controls at baseline and after 1 year of follow-up

	Baseline	Follow-up
Cases	NGT: 7 (53.8%)	NGT: 7 (53.8%)
	IGT: 4 (30.8%)	IGT: 4 (30.8%)
	CFRD: 2 (15.4%)	CFRD: 2 (15.4%)
Controls	NGT: 4 (30.8%)	NGT: 5 (38.5%)
	IGT: 7 (53.8%)	IGT: 5 (38.5%)
	CFRD: 2 (15.4%)	CFRD: 3 (23.1%)

Data are presented as count (fraction)

## Discussion

This is the first study to address the magnitude of variation on glucose tolerance following one-year treatment with CFTR modulators, by evaluating OGTT-related variables that are clinically relevant for CF patients. In fact, although the etiology of CFRD is not completely understood, the current view is that it is a multifactorial condition resulting from a combination of insulin deficiency and resistance. We used a model [19] previously adopted to quantify the insulin secretory and sensitivity defects inherent to CF compared to healthy controls [23], their dependence on sex and age [16] and their relationship to respiratory defects [24].

An observational study on LUMA/IVA-treated patients matched with historical controls was therefore performed. All cases who started treatment fulfilled the criteria required for clinical drug prescription (homozygous for Phe508del CFTR mutation and at least 12 years of age). Of those who started treatment, matching with appropriate controls with the same genotype who did not receive LUMA/IVA was possible for 13 cases.

We found that no changes occurred after treatment in β-cell glucose sensitivity, an OGTT-derived parameter capturing an important feature of insulin secretory response, representing the slope of the dose–response of insulin secretion to glucose increments. At baseline, β-cell glucose sensitivity was within the expected range for patients with CF, in reference to sex and age [16]. As previously shown, β-cell glucose sensitivity is markedly reduced compared to healthy controls [4]. Also, we had already reported, cross-sectionally, that β-cell glucose sensitivity decreases by 2% yearly. We did not have the statistical power to recognize such small changes over one-year study, but we would not consider them clinically significant. A second mechanism impairing glucose tolerance, i.e. increased insulin clearance, did not vary with the treatment and a third mechanism, insulin sensitivity, did not change either.

Finally, glucose tolerance itself, whose main determinants were not influenced by treatment as shown above, was not different in treated patients. The only significant exception was a limited increment in fasting glucose concentrations in the treated group.

Our results are in line with previous results by Thomassen [12] and Li [13]. While Misgault et al. [14] did record some improvements that we did not capture, it cannot be excluded that those changes occurred for the high variability of those parameters in CF [25].

The limited sample size may be a limitation of this study. Sample size was affected both by the focus of the present study on patients with Phe508del mutation without CFRD and by the other several inclusion criteria reported to access to LUMA/IVA therapy, as reported elsewhere [10]. To this, one should consider also that distance between glucose tolerance tests further limited the total number of patients recruitable. However, small sample size does not impact generalizability of the present findings, considering also the clear strength coming from the stringent matching procedure for a large number of confounders. Indeed, we included more than one thousand studies uniquely characterizing the determinants of glucose tolerance in CF. Also, SMD shows some imbalance among baseline covariates, excluding sex. Although arbitrary thresholds for SMD have been proposed, there is no consensus on which threshold’s value should be used [20].

As previously mentioned, the combinations lumacaftor–ivacaftor and the one subsequently developed tezacaftor–ivacaftor result in modest CFTR correction compared to wild-type CFTR. Further clinical efficacy has recently been documented with the introduction of triple therapy, i.e. combining second-generation correctors, such as VX-445 (elexacaftor) to tezacaftor–ivacaftor, in CF patients homozygous for the Phe508del CFTR mutation [26] and also in those for whom no CFTR modulators are currently applied [27].

The effects of these highly effective, disease-modifying drugs on glucose metabolism and incidence of CFRD should be promptly assessed by means of adequate prospective studies.

## References

[1] Manderson Koivula FN, McClenaghan NH, Harper AGS, Kelly C (2016). Islet-intrinsic effects of CFTR mutation. Diabetologia.

[2] Moran A, Pillay K, Becker D, Granados A, Hameed S, Acerini CL (2018). ISPAD Clinical practice consensus guidelines 2018: management of cystic fibrosis-related diabetes in children and adolescents. Pediatric Diabetes.

[3] Bell SC, Mall MA, Gutierrez H, Macek M, Madge S, Davies JC, Burgel P-R (2020). The future of cystic fibrosis care: a global perspective. Lancet Resp Med.

[4] Battezzati A, Mari A, Zazzeron L, Alicandro G, Claut L, Battezzati PM, Colombo C (2011). Identification of insulin secretory defects and insulin resistance during oral glucose tolerance test in a cohort of cystic fibrosis patients. Eur J Endocrinol.

[5] Scheuing N, Holl RW, Dockter G, Hermann JM, Junge S (2014). High variability in oral glucose tolerance among 1128 patients with cystic fibrosis: a multicenter screening study. PLoS ONE.

[6] Hayes D, McCoy KS, Sheikh SI (2014). Resolution of cystic fibrosis-related diabetes with ivacaftor therapy. Am J Respir Crit Care Med.

[7] Bellin MD, Laguna T, Leschyshyn J, Regelmann W, Dunitz J, Billings JoAnne, Moran A (2013). Insulin secretion improves in cystic fibrosis following ivacaftor correction of CFTR: a small pilot study. Pediatric Diabetes.

[8] Bessonova L, Volkova N, Higgins M, Bengtsson L, Tian S, Simard C, Konstan MW (2018). Data from the US and UK cystic fibrosis registries support disease modification by CFTR modulation with ivacaftor. Thorax.

[9] Volkova N, Moy K, Evans J, Campbell D, Tian S, Simard C, Higgins M (2020). Disease progression in patients with cystic fibrosis treated with ivacaftor: data from national US and UK registries. J Cyst Fibros.

[10] Wainwright CE, Stuart Elborn J, Ramsey BW, Marigowda G, Huang X, Cipolli M, Colombo C (2015). Lumacaftor–ivacaftor in patients with cystic fibrosis homozygous for Phe508del CFTR. N Engl J Med.

[11] Boyle MP, Bell SC, Konstan MW, McColley SA, Rowe SM, Rietschel E, Huang X, Waltz D, Patel NR, Rodman D (2014). A CFTR corrector (lumacaftor) and a CFTR potentiator (ivacaftor) for treatment of patients with cystic fibrosis who have a Phe508del CFTR mutation: a phase 2 randomised controlled trial. Lancet Resp Med.

[12] Thomassen JC, Mueller MI, Alejandre MA, Alcazar ER, van Koningsbruggen-Rietschel S (2018). Effect of lumacaftor/ivacaftor on glucose metabolism and insulin secretion in phe508del homozygous cystic fibrosis patients. J Cyst Fibros.

[13] Li A, Vigers T, Pyle L, Zemanick E, Nadeau K, Sagel SD, Chan CL (2019). Continuous glucose monitoring in youth with cystic fibrosis treated with lumacaftor-ivacaftor. J Cyst Fibros.

[14] Misgault B, Chatron E, Reynaud Q, Touzet S, Abely M, Melly L, Dominique S (2020). Effect of one-year lumacaftor-ivacaftor treatment on glucose tolerance abnormalities in cystic fibrosis patients. J Cyst Fibros: Off J Eur Cyst Fibros Soc.

[15] Scheuing NAND, Holl RWAND, Dockter GAND, Hermann JMAND, Junge SAND, Koerner-Rettberg CAND, Naehrlich LAND, Smaczny CAND, Staab DAND, Thalhammer GAND, van Koningsbruggen-Rietschel SAND, Ballmann M (2014). High variability in oral glucose tolerance among 1128 patients with cystic fibrosis: a multicenter screening study. PLoS ONE.

[16] Battezzati A, Bedogni G, Zazzeron L, Mari A, Battezzati PM, Alicandro G, Bertoli S, Colombo C (2015). Age- and sex-dependent distribution of OGTT-related variables in a population of cystic fibrosis patients. J Clin EndocrinolMetabol.

[17] Miller MR, Crapo R, Hankinson J, Brusasco V, Burgos F, Casaburi R, Coates A (2005). General considerations for lung function testing. Eur Respir J.

[18] Quanjer PH, Stanojevic S, Cole TJ, Baur X, Hall GL, Culver BH, Enright PL (2012). Multi-ethnic reference values for spirometry for the 3–95 yr age range: the global lung function 2012 equations. Eur Res J.

[19] Mari A, Tura A, Gastaldelli A, Ferrannini E (2002). Assessing insulin secretion by modeling in multiple-meal tests. Diabetes.

[20] Nguyen T-L, Collins GS, Spence J, Jean-Pierre Daurès PJ, Devereaux PL, Le Manach Y (2017). Double-adjustment in propensity score matching analysis: choosing a threshold for considering residual imbalance. BMC Med Res Methodol.

[21] Ho D, Imai K, King G, Stuart E (2007). Matching as nonparametric preprocessing for reducing model dependence in parametric causal inference. Political Analysis.

[22] R Core Team (2019) R: a language and environment for statistical computing. Vienna, Austria: R foundation for statistical computing. https://www.R-project.org/.

[23] Ho, Daniel E, Kosuke Imai, Gary King, Elizabeth A. Stuart (2011) MatchIt: Nonparametric preprocessing for parametric causal inference. J Stat Software 42 (8): 1–28. http://www.jstatsoft.org/v42/i08/.

[24] Colombo C, Alicandro G, Gambazza S, Mileto P, Mari A, Grespan E, Nazzari E, Russo MC, Battezzati A (2019). Ventilation inhomogeneity is associated with OGTT-derived insulin secretory defects in cystic fibrosis. Pediatr Pulmonol.

[25] Ballmann M, Prinz N, Glass A, Holl R (2020). Comment on “effect of one-year lumacaftor-ivacaftor treatment on glucose tolerance abnormalities in cystic fibrosis patients”. J Cyst Fibros.

[26] Heijerman HGM, McKone EF, Downey DG, Van Braeckel E, Rowe SM, Tullis E, Mall MA (2019). Efficacy and safety of the elexacaftor plus tezacaftor plus ivacaftor combination regimen in people with cystic fibrosis homozygous for the f508del mutation: a double-blind, randomised, phase 3 trial. The Lancet.

[27] Middleton PG, Mall MA, Dřevínek P, Lands LC, McKone EF, Polineni D, Ramsey BW (2019). Elexacaftor–tezacaftor–ivacaftor for cystic fibrosis with a single Phe508del allele. N Engl J Med.

